# Trapped Lung: A Rare Complication of Rheumatoid Arthritis

**DOI:** 10.7759/cureus.88213

**Published:** 2025-07-18

**Authors:** Aditya K Adhikarla, Asya Azad, Muhammad A Fazal

**Affiliations:** 1 Internal Medicine, Manchester Royal Infirmary, Manchester, GBR; 2 General Internal Medicine, Tameside General Hospital, Manchester, GBR; 3 Respiratory Medicine, Tameside General Hospital, Manchester, GBR

**Keywords:** auto immune, recurrent hydropneumothorax, rheumatoid lung disease, rheumatoid nodule, trapped lung

## Abstract

Trapped lung is a rare complication of rheumatoid arthritis (RA), often resulting from chronic pleural inflammation and fibrosis. It presents as a non-expandable lung with persistent pleural separation despite drainage. We report a 67-year-old woman with seropositive RA and multiple co-morbidities, who presented with chronic left-sided hydro-pneumothorax to the respiratory clinic. Imaging showed pleural thickening, lung nodules, and pleural tethering in addition to the pneumothorax. Due to poor functional reserve, multiple lung nodules and stable symptoms, she was managed conservatively after multidisciplinary input. Trapped lung should be considered in RA patients with chronic pleural effusion and non-resolving pneumothorax. Diagnosis relies on imaging and clinical picture. There are multiple treatment options, with surgical decortication as the definitive modality. Management is planned according to symptom burden and patient suitability for invasive intervention.

## Introduction

Rheumatoid arthritis (RA), although primarily recognised as a chronic inflammatory joint disorder, is well known for its multi-system involvement, with pulmonary manifestations representing a significant cause of morbidity. Pulmonary involvement in RA is diverse and may include interstitial lung disease, pleural effusions, pleuritis, pulmonary rheumatoid nodules, airway disease (such as bronchiolitis and bronchiectasis) and pulmonary vasculitis [1].

Trapped lung or non-expandable lung is a rare pleural complication of RA. It is characterised by the lung’s inability to expand due to the formation of a fibrous peel encasing the visceral pleura, often as a sequela of chronic pleural inflammation. The diagnosis should be suspected in patients with a history of pleural disease and chronic pleural effusion. It can be established through contrast-enhanced computed tomography (CT), which reveals visceral pleural thickening, or by direct visualisation of a fibrous pleural rind during video-assisted thoracoscopic surgery or medical thoracoscopy. Pleural manometry, while diagnostically useful, is time-consuming and seldom utilised in routine practice due to limited accessibility and the expertise required. Management ranges from observation to more invasive interventions, such as indwelling pleural catheters (IPCs), fibrinolysis, and surgical decortication [2-4].

We present a case of trapped lung in a patient with longstanding RA, highlighting the diagnostic complexity and management considerations.

## Case presentation

A 67-year-old Caucasian woman with a longstanding history of seropositive RA (anti-CCP 235 and rheumatoid factor 148) and chronic obstructive pulmonary disease (COPD), both present for over two decades, was reviewed in the respiratory clinic of a district general hospital in August 2024. Her past medical history was also notable for osteoarthritis, heart failure, hypertension, and type 2 diabetes mellitus. An NT-proBNP level measured in early 2025 was elevated at 8,946 ng/L (reference range: 0-400 ng/L), and an echocardiogram from the same period showed an estimated left ventricular ejection fraction of 40-45%, with evidence of impaired systolic and diastolic function. Her RA was well controlled under rheumatology care and managed with abatacept, oral hydroxychloroquine, and a tapering course of oral corticosteroids. She also had a history of recurrent bilateral hydropneumothoraces and pleural effusions.

**Figure 1:**
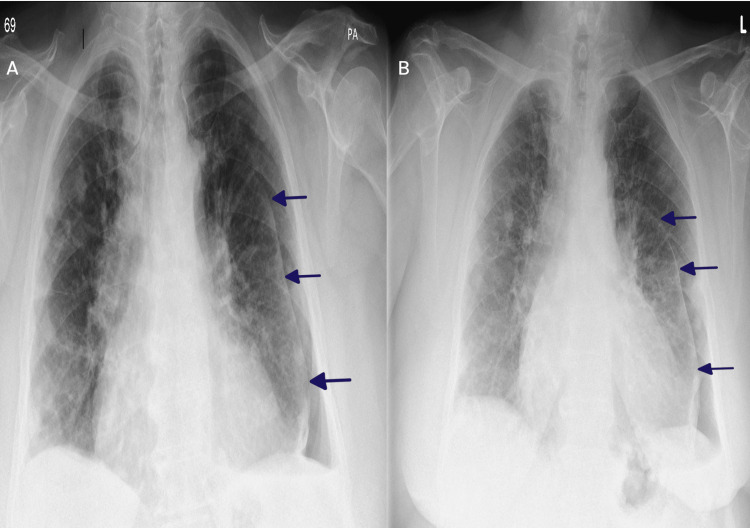
Serial chest X-rays (posteroanterior view) demonstrating left-sided hydropneumothorax. A) Initial image from the respiratory clinic showing a left-sided hydropneumothorax. B) Follow-up image from the pleural clinic showing a marginal increase in the pneumothorax size Blue arrow: Lung margin

On clinical examination, breath sounds were markedly reduced over the left hemithorax, with no additional sounds. Her oxygen saturation was 90% on room air, with a respiratory rate of 19 breaths per minute. A chest radiograph performed in the clinic revealed a left-sided hydropneumothorax (Figure 1A). Given her stable exertional dyspnoea over the past year and absence of acute respiratory symptoms, this was interpreted as a chronic finding. In the context of her longstanding RA and a history of chronic pleural effusion, a trapped lung was suspected. Notably, she had undergone a lung nodule biopsy two years earlier, with histopathology confirming granulomatous nodules with central fibrinoid necrosis, consistent with a rheumatoid aetiology.

She had previously undergone multiple pleural aspirations, all of which were negative for malignancy and infection. A pleural fluid sample from 2022 demonstrated foamy macrophages and multinucleate giant cells consistent with rheumatoid pleuritis. In light of the current findings, she was referred for an urgent contrast-enhanced CT of the thorax and scheduled for review in the pleural clinic within two weeks, including a thoracic ultrasound and consideration of therapeutic aspiration.

At the scheduled review in the pleural clinic, thoracic ultrasound demonstrated left-sided pleural thickening with minimal residual effusion, which was not amenable to aspiration. A repeat chest X-ray (Figure 1B) showed a marginal increase in the size of the pneumothorax. Given this, along with a resting oxygen saturation of 85% on room air, she was admitted to the respiratory ward for further evaluation and management. During her inpatient stay, she received oxygen therapy via nasal cannula at two litres per minute to maintain target saturations of 88-92%, in view of her underlying COPD with chronic carbon dioxide retention. Routine blood investigations showed no evidence of active infection. Her overall COPD management was reviewed during admission. She was on maintenance inhaler therapy with formoterol fumarate, glycopyrronium bromide, and budesonide (Trixeo, two puffs twice daily), along with salbutamol as a short-acting reliever. Her inhaler technique was reviewed and found to be appropriate.

**Figure 2:**
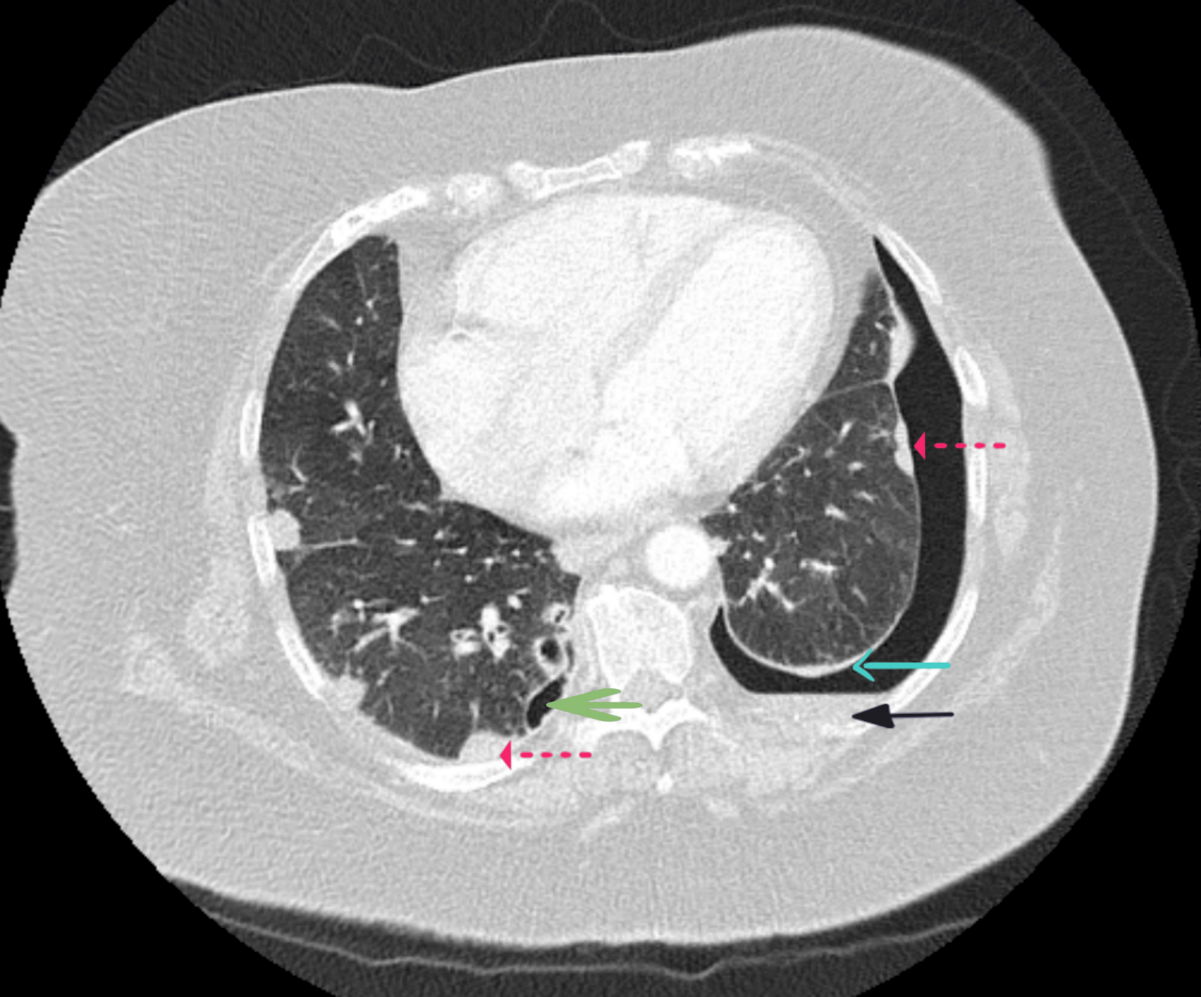
Axial view of contrast-enhanced CT thorax demonstrating a 13 mm left-sided hydropneumothorax, an old loculated right-sided pneumothorax, pleural thickening and subpleural rheumatoid nodules CT: Computed tomography Solid blue arrow: Pleural thickening; Solid black arrow: Pleural effusion; Solid green arrow: Loculated pneumothorax; Dotted red arrow: Rheumatoid nodule

A contrast-enhanced CT of the thorax (Figure 2), performed during admission in August 2024, confirmed a 13 mm left-sided pneumothorax, an old loculated right-sided pneumothorax, a small left-sided pleural effusion, pleural thickening, pleural tethering and multiple bilateral pulmonary nodules. These findings were consistent with a trapped lung, characterised by persistent pleural thickening and tethering of the left lung. An attempt was made to insert a chest drain; however, the procedure was aborted due to technical difficulty and the limited anticipated clinical benefit.

Owing to her age, significant comorbidities including heart failure, COPD, and type 2 diabetes mellitus, poor baseline functional status, and stable respiratory symptoms, she was not considered a suitable candidate for surgical decortication. Following discussion with the regional thoracic surgery team, a conservative management approach was advised. A key factor influencing this decision was the presence of subpleural rheumatoid nodules, which carry a recognised risk of rupture during decortication and could potentially precipitate a pneumothorax.

**Figure 3:**
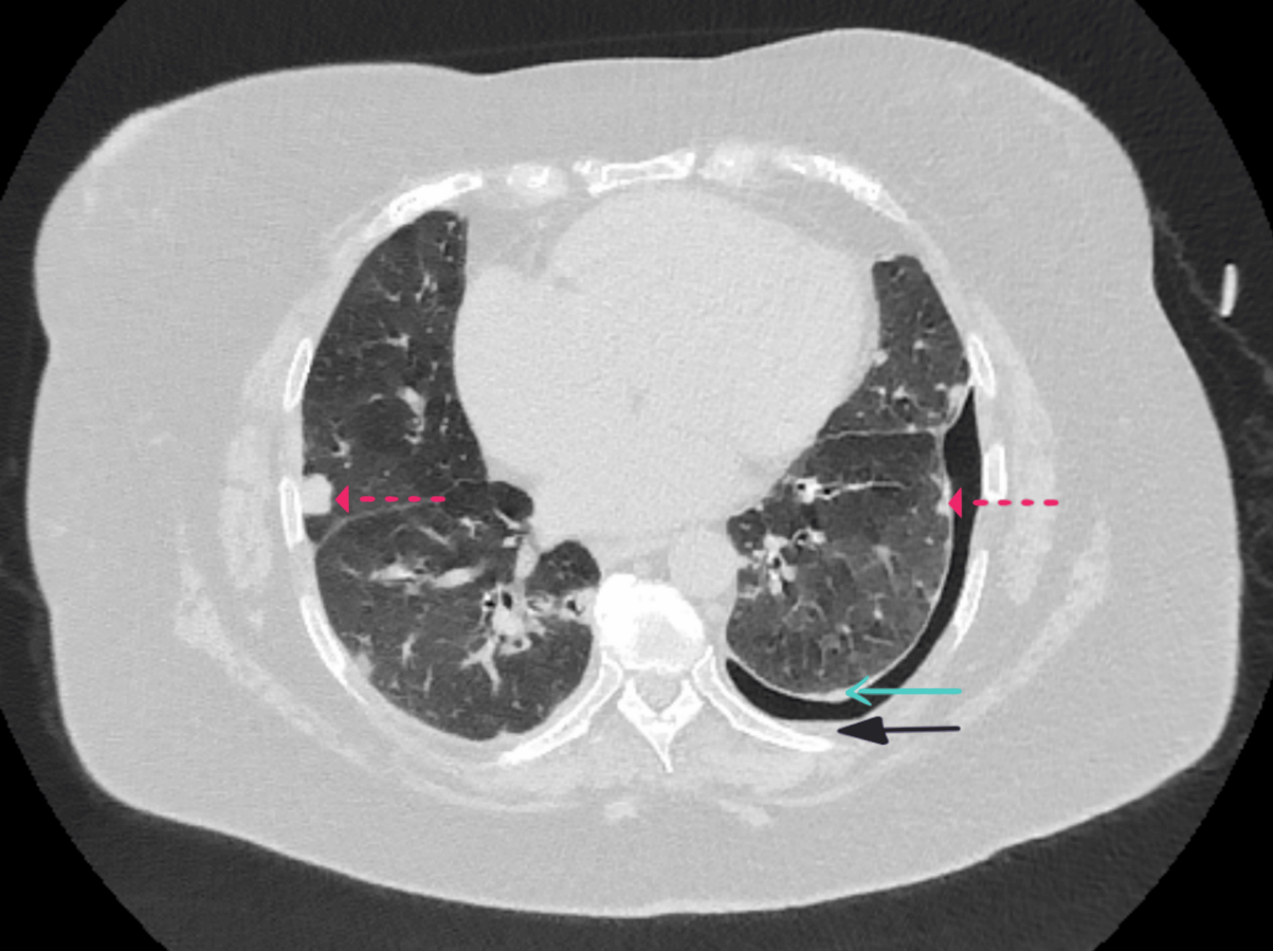
Axial view of repeat contrast-enhanced CT thorax demonstrating a 12 mm left-sided pneumothorax, minimal pleural effusion, pleural thickening, and stable subpleural rheumatoid nodules CT: Computed Tomography Solid blue arrow: Pleural thickening; Solid black arrow: Pleural effusion; Dotted red arrow: Rheumatoid nodule

The patient remained hospitalised for over four weeks and was discharged in early October 2024 with a management plan centred on symptom control and outpatient follow-up with rheumatology and respiratory services. A repeat contrast-enhanced CT thorax was performed in October 2024, with a follow-up appointment arranged to review the results. She was subsequently seen in the respiratory clinic in November 2024, where she was clinically stable with an oxygen saturation of 90% on room air. Repeat imaging (Figure 3) demonstrated a marginal reduction in the left-sided pneumothorax to 12 mm and minimal pleural fluid. 

Considering her stable condition and marginal reduction in pneumothorax size seen on repeat imaging, she was transitioned to a patient-initiated follow-up pathway.

## Discussion

A trapped lung refers to the inability of the lung to fully re-expand within the thoracic cavity due to the presence of a fibrous peel over the visceral pleura that restricts expansion. The diagnosis is typically established in the setting of a chronic pleural effusion, supported by characteristic radiological findings [3]. In this case, the patient’s longstanding history of seropositive RA and recurrent pleural effusions, along with CT evidence of pleural thickening and tethering (Figure 2), supported the diagnosis of a trapped lung.

Management decisions are guided by the severity of symptoms, baseline functional status and the patient’s overall clinical picture. Treatment options include IPCs, pleuroperitoneal shunting, intrapleural fibrinolytic therapy and surgical decortication. Among these, surgical decortication remains the definitive intervention in suitable candidates. Multidisciplinary input involving respiratory physicians, thoracic surgeons, and interventional radiologists is essential to guide appropriate management [2,5]. In this case, early involvement of the regional thoracic surgery team was sought to evaluate therapeutic options.

The patient remained clinically stable, without worsening dyspnoea, and had a poor baseline functional reserve, requiring assistance with daily activities. A prior lung nodule biopsy had confirmed the presence of subpleural rheumatoid nodules. Although uncommon, these nodules can rupture into the pleural space, potentially resulting in complications such as pneumothorax or bronchopleural fistula [6]. The presence of multiple nodules raised concern for intraoperative risk during potential decortication. Owing to her stable clinical condition, significant comorbidities, limited functional reserve, and the procedural risks, conservative management was deemed the most appropriate approach following multidisciplinary review.

At her follow-up appointment, a repeat contrast-enhanced CT (Figure 3) of the thorax demonstrated a marginal reduction in the size of the left-sided pneumothorax, and she remained clinically stable. This further supported the decision to continue with conservative management.

## Conclusions

This case underscores the importance of maintaining a high index of suspicion for trapped lung in patients with long-standing RA who present with recurrent pleural effusions and characteristic radiological features. It also highlights the value of early multidisciplinary team involvement to guide individualised management decisions, considering symptom burden, functional capacity, and comorbid conditions. Furthermore, the presence of subpleural rheumatoid nodules may limit the feasibility of invasive interventions due to an increased risk of procedural complications.
